# Controlled *in vivo* Bone Formation and Vascularization Using Ultrasound-Triggered Release of Recombinant Vascular Endothelial Growth Factor From Poly(D,L-lactic-co-glycolicacid) Microbubbles

**DOI:** 10.3389/fphar.2019.00413

**Published:** 2019-04-24

**Authors:** Yong Gong, Songjian Li, Wei Zeng, Jianing Yu, Yan Chen, Bo Yu

**Affiliations:** ^1^ Department of Orthopedics, Zhujiang Hospital, Southern Medical University, Guangzhou, China; ^2^ Ultrasound Medical Center, Zhujiang Hospital, Southern Medical University, Guangzhou, China; ^3^ The First School of Clinical Medicine, Southern Medical University, Guangzhou, China

**Keywords:** bone defect, vascular endothelial growth factor, angiogenesis, ultrasound, osteogenesis

## Abstract

Bone defects are challenging to treat in musculoskeletal system due to the lack of vascularization. Biomaterials with internal vascularization ability and osteoinduction bioactivity are promising strategies for orthopedic applications. Vascular endothelial growth factor (VEGF) has been widely used for angiogenesis and osteogenesis. Here, we developed VEGF-loaded PLGA microbubbles (MBs) for improvement of angiogenesis and osteogenesis in bone defect repair in combination with ultrasound-targeted microbubble destruction (UTMD). Release profile showed UTMD promoted the burst release of VEGF from PLGA MBs. We subsequently investigated the combination of ultrasound application with VEGF MBs for *in vitro* osteogenesis. The results demonstrated that the expression of osteogenesis-related genes and calcium deposits were increased by VEGF MBs in combination of UTMD. Micro-computed tomography (micro-CT) and histological analysis were conducted 4 and 8 weeks post-surgery. *In vivo* results show that VEGF MBs in combination of UTMD could significantly enhance new bone formation and vascular ingrowth at the defect site in a rat calvarial defect model. In summary, VEGF MBs in combination of UTMD could augment bone regeneration and vascularization at calvarial bone defects and hold huge potential for clinical translation.

## Introduction

The clinical treatment of large bone defects is still a great challenge (Tang et al., 2016; Dang et al., 2017). Early vascularization at the defect area is essential for bone formation (Spiller et al., 2015). Since angiogenesis is closely related to bone regeneration, various growth factors including fibroblast growth factor 2 (FGF2), transforming growth factor (TGF-β), and vascular endothelial growth factor (VEGF) play an important role in neovascularization and endochondral ossification (Florencio-Silva et al., 2015; Filipowska et al., 2017). Currently, treatments for angiogenesis aim to localized concentration and continuous usage of growth factors. The release of the drug system allows concentrated, low-dose angiogenic factors to act for a long time to promote tissue regeneration. Various natural, synthetic, and complex materials have been used as release carriers for angiogenic growth factors (Freudenberg et al., 2015). VEGF is a key regulator of angiogenesis and also plays an important role in osteogenesis (Gerber et al., 1999; Hu and Olsen, 2016). Localized VEGF delivery has proven effective for osteogenesis in many studies (Kempen et al., 2009; García et al., 2016).

Microbubbles (MBs) are widely used as ultrasound contrast agents, which also have potential therapeutic applications (Lawrie et al., 2000; Prentice et al., 2005). Drugs and genes can be incorporated in the bubble construct. Recently, poly(D,L-lactic-co-glycolicacid) (PLGA) MBs have been reported as drug and gene carriers in different medical and biological applications (Sirsi and Borden, 2009; Formiga et al., 2010). However, there are some limitations in the conventional drug-loaded MBs for controlled release, such as low local concentration and unable to target release (Hernot and Klibanov, 2008). Ultrasound targeted microbubble destruction (UTMD) has been demonstrated as a new promising strategy for non-invasive, targeted drug and gene delivery (Bekeredjian et al., 2006; Tinkov et al., 2009; Chen and Hwang, 2013). Various studies have developed MBs loaded with antitumor genes and drugs successfully in combination with ultrasound for treatment of tumors in animal models (Kiessling et al., 2012; Zhao et al., 2016). However, using VEGF-loaded microbubbles with UTMD for the treatment of bone defects have not been reported yet.

Therefore, in this study, we prepared PLGA MBs containing the angiogenic cytokine VEGF that burst release VEGF in response to ultrasound exposure. We fabricated MBs loaded with or without VEGF using the PLGA 50/50 and PLGA 75/25 copolymers and investigated the release profile, *in vitro* and *in vivo* osteogenesis and angiogenesis capacity.

## Materials and Methods

### Materials

Recombinant human VEGF (rhVEGF165, Sf21-derived) and Quantikine VEGF Elisa kit were purchased from R&D Systems (Minneapolis, MN, USA). Poly(dl-lactide-co-glycolide) (PLGA; *L*/*G* = 50/50, *MW*: 40,000–75,000; *L*/*G* = 75/25, *MW*: 90,000–120,000) was provided by Jinan Daigang Biomaterials (Jinan, China). Polyethylene glycol (PEG; *MW*: 400), bovine serum albumin (BSA), and sodium azide were purchased from Sigma-Aldrich (USA). Poly(vinyl alcohol) (PVA) (*MW*: 125,000) was obtained from Polysciences, Inc. (Warrington, USA). Rabbit polyclonal to CD31 (ab28364)) was supplied by Abcam (Cambridge, MA, USA).

### Preparation of PLGA MBs

PLGA MBs were fabricated by the emulsion solvent evaporation method. Briefly, 1.0 g PLGA (50/50, 75/25) was dissolved in 10 ml dichloromethane. A 100-μg VEGF and 5-μl PEG 400 dissolved in 200 μl of water were injected into PLGA solution. For preparation of BSA-loaded MBs, 5 mg of BSA and 5 μl of PEG 400 dissolved in 200 μl of water were injected into PLGA solution. Subsequently, mixed solution was injected into 100 ml of 1% PVA solution, resulting in a multiple emulsion. The multiple emulsion was stirred at 1,000 rpm for 10 h, then collected, and lyophilized. Finally, the MBs were resuspended in 1 ml of ultrapure water, frozen at −80°C, lyophilized, and stored at 4°C. MBs were sterilized by cobalt 60 (^60^Co) irradiation.

### Characterization of MBs

PLGA MBs were observed by scanning electron microscope (SEM, JSM-7001F, Japan). To evaluate the release profile, 3 mg of VEGF-loaded or BSA-loaded 75/25 and 50/50 microbubbles (n = 3) were suspended in 2.0 ml of 0.1 M phosphate buffer (pH 7.4), containing 0.1% BSA and microbiologically preserved with sodium azide. The samples were maintained in rotating vials at 37°C. At 2 and 7 days, sample tubes were exposed to ultrasound radiation. The parameters of US exposure were as follows: frequency, 1 MHz; intensity, 2 W/cm^2^; duty cycle, 50%; pulse recurrent frequency, 100 Hz; and duration, 5 min. At scheduled time intervals, sample tubes were centrifuged (25,000 × *g*, 15 min) and the supernatants of 100 μl were harvested and frozen at −80°C. The amounts of released VEGF were quantified using ELISA kits, and released BSA was quantified using a BCA Protein Assay Kit. The *in vitro* ultrasound imaging of MBs was conducted using Visual Sonic 2100 with a MS-250 transducer (VisualSonics, Canada). Blank MBs and VEGF-MBs were dispersed in PBS at 1 × 10^6^/ml, and PBS was used as a control.

### Alizarin Red S Staining

Alizarin red S staining was determined as described previously (Chen et al., 2017). Briefly, bone marrow stromal cells (BMSCs) at different groups were relatively cultured in osteogenic induction medium at a density of 1.0 × 10^5^ cells/well. After 21 days, cells were fixed with 4% (w/v) paraformaldehyde and then stained with 1% alizarin red S (Biochem, Shanghai, China) for 30 min at 25°C with gentle agitation. The cells were gently rinsed with ultrapure water for three times and observed under microscope (Olympus, Tokyo, Japan).

### Real-Time Quantitative PCR (RT-qPCR)

After 10 days of co-culture, total RNA was isolated by lysis in TRIzol (Invitrogen Inc., Carlsbad, CA, USA). Total RNA was reverse-transcribed into cDNA from 1.0 μg of the RNA using ReadyScript cDNA Synthesis Mix (Sigma). The mRNA levels of osteogenic-specific genes including osteocalcin (OCN), alkaline phosphatase (ALP), and Runx2 were assessed by RT-qPCR using SYBR Green Master (Roche). β-actin was amplified as an internal control.

### Animal Experiments

Ethical approval was obtained from Southern Medical University Institutional Animal Care and Use Committee. Twenty-four male SD rats (190–240 g) were used for this experiment. The animals were housed under standard conditions with free access to food and water. The experimental groups included blank defect (control, *n* = 6), blank MB (MB, *n* = 6), VEGF-loaded MB (VEGF-MB, *n* = 6), and VEGF-loaded MB in combination with UTMD (VEGF-MB + US, *n* = 6). Under the general anesthesia of ketamine (100 mg/kg bodyweight) and xylazine (10 mg/kg bodyweight), the scalps were exposed. A 5-mm diameter bilateral calvarial defect was created in each rat using a dental bur. 75/25 and 50/50 blank MBs, and VEGF-loaded PLGA MBs were mixed with thiolated chitosan/hydroxyapatite thermo-sensitive hydrogel as we previously described (Liu et al., 2014). Hydrogels were implanted into the defects. After surgery, skin was sutured with a 4–0 silk suture. At 2 and 10 days post-surgery, skulls were exposed to ultrasound for 20 min at the same parameter with *in vitro* study.

### Micro-CT Analysis

After harvesting, the skulls at 4 and 8 weeks post-operatively, the specimens were immediately fixed in 10% (v/v) neutral buffered formalin for 48 h. Specimens were scanned at 9 μm resolution for undecalcified samples using an advanced micro-CT instrument (ZKKS-MC-Sharp-IV, Zhongke Kaisheng Bio, Inc.) with scanning parameters of 50 kV, 200 mA and a 0.5-mm aluminum filter. Bone density measurement was performed based on the ROI determined in each sample slice. Trabecular number (Tb.N) was evaluated.

### Immunohistochemistry and Histomorphometry

Specimens were decalcified in neutral 10% EDTA solution for 2 weeks at room temperature. After decalcification, 5-μm thick serial slices were sectioned and further stained with anti-CD31 (1:600 dilution; Abcam, Cambridge, MA, USA), respectively, at 4°C overnight. Samples were subsequently incubated with goat anti-rabbit second antibody conjugated with HRP (Boster Company of Biotechnology, China). The imagines of stained specimens were visualized with microscopy. The sections were also stained with hematoxylin and eosin (HE) staining.

### Statistics

Descriptive statistics were used to determine group means and standard deviations. Quantitative data were statistically analyzed using the student’s *t*-test analysis. Statistically, significance was set at *p* < 0.05.

## Results

### Characterization of PLGA MBs

**Figure 1:**
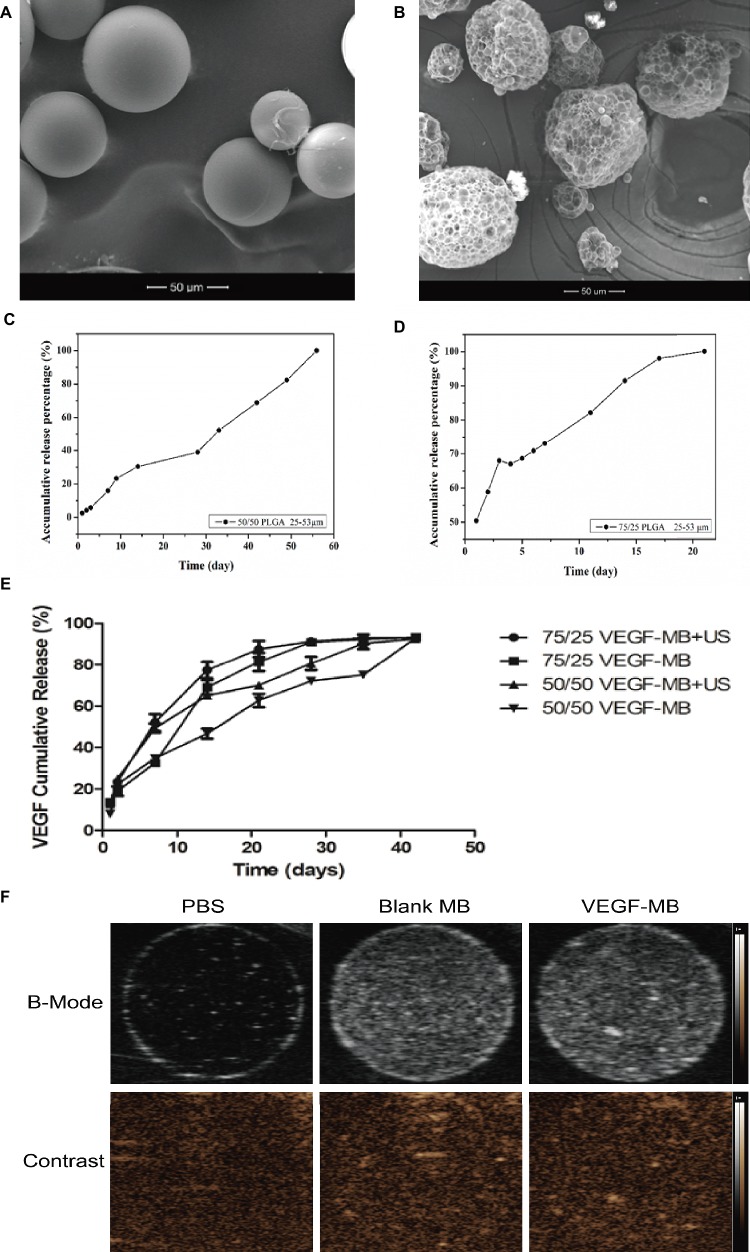
Characterization of PLGA MBs. **(A)** SEM of blank PLGA MBs. **(B)** SEM of VEGF-loaded PLGA MBs. (Scale bar = 50 μm). **(C,D)** Release profile of BSA from 50/50 and 75/25 PLGA MBs. **(E)** Release profile of VEGF from 50/50 and 75/25 PLGA MBs with or without UTMD. **(F)** Ultrasound images of blank PLGA MBs and VEGF-loaded PLGA MBs dispersed in PBS. Upper panel, B-mode images; bottom panel, contrast-mode images.

SEM analysis showed morphologically intact and smooth surface in blank MBs (Figure 1A). Blank MBs showed a fairly uniform distribution and good shelf stability. After loaded with VEGF, the surfaces of MBs were coarser (Figure 1B). The average diameter of MBs was about 50 μm. The concentration of MBs was 1 × 10^7^/ml. For *in vitro* release investigation, 50/50 and 75/25 BSA-loaded PLGA MBs showed different release behaviors. The release of BSA from 50/50 PLGA MBs was maintained over 60 days, while 72/25 PLGA MBs exhibited a release period of 20 days (Figures 1C,D). Figure 1E shows the release profile of VEGF from MBs in PBS (pH 7.4) at 37°C. VEGF was incessantly released from the MBs without US radiation. After US exposure, the burst release was observed in the MBs. US exposure promoted the burst release of VEGF. Figure 1F shows the signal of blank MBs and VEGF-MBs can be visualized by ultrasound imaging.

### *In vitro* Osteogenesis

**Figure 2:**
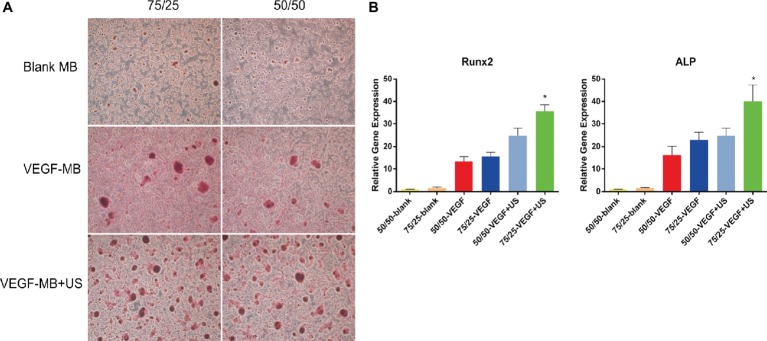
*In vitro* osteogenesis of PLGA MBs. **(A)** Alizarin red S staining of BMSCs cultured with blank MB, VEGF-MB with or without UTMD for 21 days. **(B)** RT-qPCR analysis of osteogenesis-associated genes Runx2 and ALP expression. *p < 0.05.

The *in vitro* osteogenesis was investigated through alizarin red S staining 21 days after the BMSCs were cultured with these MBs. 75/25 and 50/50 blank MB cultured alone showed almost no positive staining, 75/25 and 50/50 VEGF-MB showed positive staining, while 75/25 VEGF-MB combined with US group presented the most significantly positive staining (Figure 2A). RT-qPCR analysis revealed that the expression of osteogenesis-associated genes OCN and ALP was highest in 75/25 VEGF-MB with ultrasound exposure (Figure 2B, *p* < 0.05).

### UTMD Delivery of VEGF Promoted Bone Defect Repair

**Figure 3:**
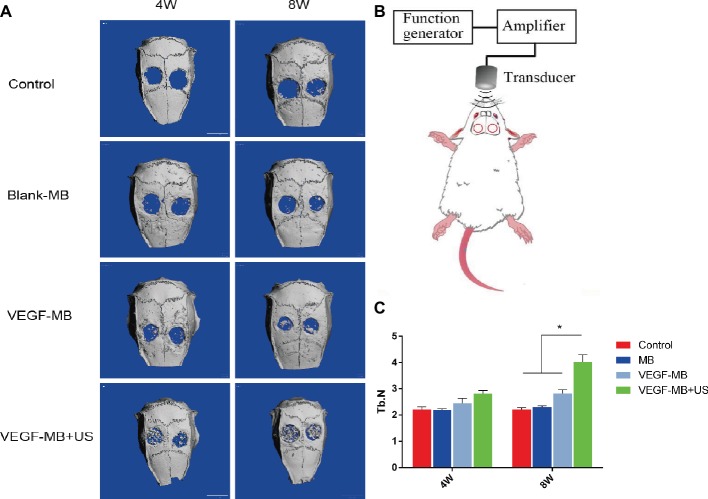
Evaluation of calvarial bone defect repair *in vivo*. **(A)** Micro-CT analysis of skulls 4 and 8 weeks post-surgery. **(B)** Schematic representation of UTMD for *in vivo* investigation. **(C)** Quantitive analysis of trabecular number 4 and 8 weeks post-surgery. *p < 0.05.

**Figure 4:**
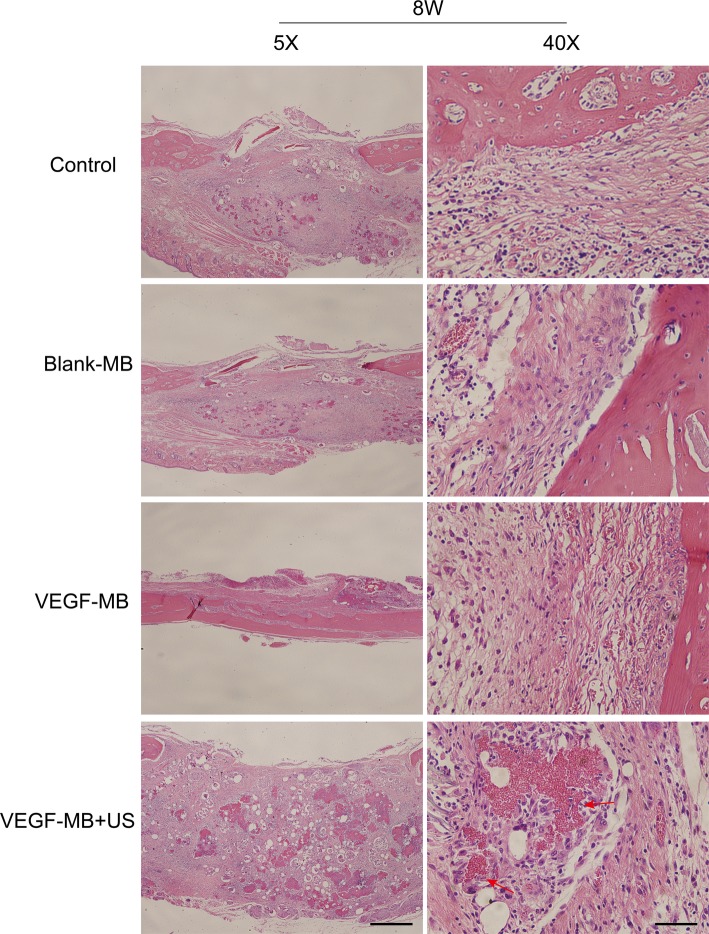
Histological assessment of bone regeneration at 8 weeks after surgery by H&E staining. Red arrow represents new vessels formation (Scale bar = 400 and 50 μm).

**Figure 5:**
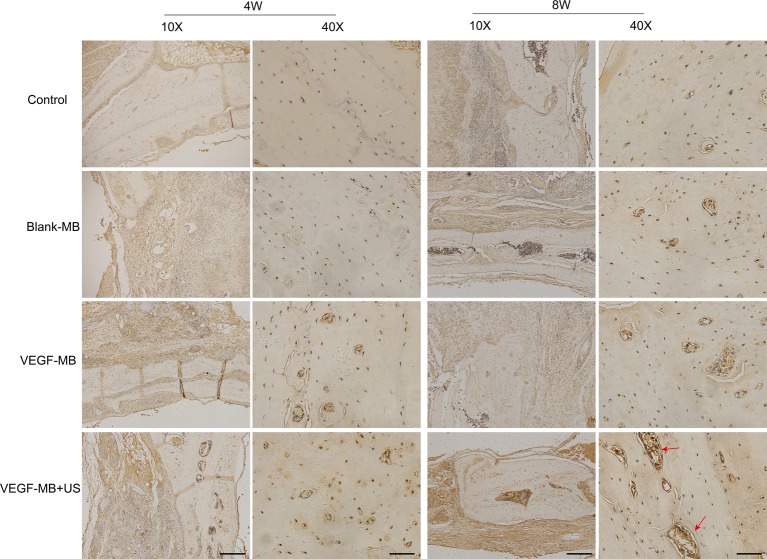
Histological assessment of bone regeneration at 4 and 8 weeks after surgery by anti-CD31 immunohistochemistry staining. Red arrow represents new vessels formation. (Scale bar = 200 and 50 μm).

MB and VEGF-MB were mixed with thiolated chitosan/hydroxyapatite thermo-sensitive hydrogel. MBs were distributed in the hydrogel. The hydrogel was free flowing liquid at room temperature, but formed solid-like gel when heated to 37°C. We next investigated the effect of VEGF-MB and UTMD on rat calvarial defect repair at 4 and 8 weeks (Figure 3B). The micro-CT imaging performed at 4 and 8 weeks revealed no bone repair in the control group and blank MB (Figure 3A). Appreciable new bone formation and ingrowth occurred in the repaired area in the VEGF-MB group at 4 and 8 weeks. The VEGF-MB + US group showed most significant new bone formation among these groups at 4 and 8 weeks. According to micro-CT analysis, the trabecular number (Tb.N) was significantly higher in VEGF-MB + US group (Figure 3C) (*p* < 0.05). HE staining was performed after 8 weeks to further verify the repair effect. No new bone formation was observed in control or blank-MB group, but fibrous tissues appeared. The VEGF-MB group showed a small number of new bone formation. Massive bone formation was found in the repaired area in the VEGF-MB + US group (Figure 4). Immunohistochemical staining was also conducted to assess CD31 expression after 4 and 8 weeks. CD31 staining revealed slight positive staining in the control and blank-MB groups, while VEGF-MB group resulted in the modern density of new blood vessels (Figure 5). The VEGF-MB + US group showed most significant positive staining, suggesting that this group exhibits the best osteogenesis capacity and bone repair ability.

## Discussion

Large or segmental bone defects caused by trauma, tumor, and inflammation have always been a difficult problem in clinical treatment (El-Rashidy et al., 2017). Injectable bone tissue engineering materials can be injected into the bone defect by minimally invasive methods. It can also carry and release growth factors and drugs. In this study, we designed VEGF-loaded microbubbles so that microbubbles could carry VEGF protein and locally burst release when conducting UTMD, leading to drug accumulation in bone defect area and enhanced repair effect. The repair and treatment of bone defects has always been one of the important problems in the clinical. The rate of autologous blood vessel growth is about tens of micrometers per day, especially for large-area bone defects, which is not enough to vascularize the entire implanted tissue (Padilla et al., 2017). It is the key factor to solve the problem of insufficient blood supply in the defect site and promote the bone repair. Early vascularization is currently the most direct and effective method for vascularizing bone substitutes (Zhang et al., 2017). However, due to the deficiencies of multiple operations, this method is limited to the repair of small-area bone defects and soft tissue injuries.

VEGF is recognized as one of the most potent cytokines in inducing angiogenesis, also directly related to bone formation (Duan et al., 2016; Simons et al., 2016). In the inflammation phase during bone repair, VEGF is accumulated in the hematoma after bone injury (Loi et al., 2016). In endochondral bone formation during bone repair, VEGF improves the migration of osteoblastic cells and induces cartilage formation (Dai and Rabie, 2007). Various reports have investigated that VEGF affects bone repair and regeneration and developed systems for local delivery of VEGF (Martino et al., 2015; Schumacher et al., 2017; Sharmin et al., 2017). PLGA-coated β-TCP scaffolds containing VEGF were developed to deliver VEGF for bone repair (Khojasteh et al., 2016). The data showed the scaffolds with VEGF, presenting most significant bone regeneration *in vitro*. VEGF-loaded hydrogels exhibited significantly increased vascularization and bone formation in segmental bone defect animal models (García et al., 2016). In this study, we found that VEGF-loaded MB could increase osteogenesis both *in vitro* and *in vivo*, which was consistent with these studies.

Ultrasound-targeted microbubble destruction (UTMD) is a novel method for drug and gene transfection based on acoustic cavitation. UTMD can release the drug locally and achieve the goal of targeted therapy in animal models (Chen et al., 2006; Kopechek et al., 2015; Zhao et al., 2016). UTMD allows the spatiotemporal target release of encapsulated VEGF in the local bone defect area. In this study, we conducted UTMD after VEGF-loaded MB implanted into rat calvarial defects. We found that UTMD promoted the burst release of VEGF and presented most significant bone regeneration *in vivo*.

In conclusion, we demonstrated the use of VEGF-loaded MB for enhancing calvarial defects by UTMD burst release. Results showed that VEGF-loaded MB combined with UTMD promoted osteogenesis *in vitro* and enhanced bone repair *in vivo* compared with those groups without UTMD treatment.

## Ethics Statement

This study was carried out in accordance with the recommendations of Guide for the Care and Use of Laboratory Animals, the Southern Medical University Institutional Animal Care and Use Committee. The protocol was approved by the Southern Medical University Institutional Animal Care and Use Committee.

## Author Contributions

YG, YC, and BY designed the experiment. YG, JY, WZ, and SL performed the experiment. YG investigated the study and wrote the manuscript. BY acquired funding, contributed to resources, and supervised the study.

### Conflict of Interest Statement

The authors declare that the research was conducted in the absence of any commercial or financial relationships that could be construed as a potential conflict of interest.

The reviewer CC declared a past co-authorship with one of the authors with the author BY to the handling editor.
